# Breast Cancer Detection Using Convoluted Features and Ensemble Machine Learning Algorithm

**DOI:** 10.3390/cancers14236015

**Published:** 2022-12-06

**Authors:** Muhammad Umer, Mahum Naveed, Fadwa Alrowais, Abid Ishaq, Abdullah Al Hejaili, Shtwai Alsubai, Ala’ Abdulmajid Eshmawi, Abdullah Mohamed, Imran Ashraf

**Affiliations:** 1 Department of Computer Science & Information Technology, The Islamia University of Bahawalpur, Bahawalpur 63100, Pakistan; 2 Fatima Jinnah Medical University, Lahore 54000, Pakistan; 3 Department of Computer Sciences, College of Computer and Information Sciences, Princess Nourah Bint Abdulrahman University, Riyadh 11671, Saudi Arabia; 4 Computer Science Department, Faculty of Computers & Information Technology, University of Tabuk, Tabuk 71491, Saudi Arabia; 5 Department of Computer Science, College of Computer Engineering and Sciences in Al-Kharj, Prince Sattam Bin Abdulaziz University, Al-Kharj 11942, Saudi Arabia; 6 Department of Cybersecurity, College of Computer Science and Engineering, University of Jeddah, Jeddah 21959, Saudi Arabia; 7 Research Centre, Future University in Egypt, New Cairo 11745, Egypt; 8 Department of Information and Communication Engineering, Yeungnam University, Gyeongsan 38541, Republic of Korea

**Keywords:** breast cancer prediction, healthcare, deep convoluted features, ensemble learning

## Abstract

**Simple Summary:**

This paper presents a breast cancer detection approach where the convoluted features from a convolutional neural network are utilized to train a machine learning model. Results demonstrate that use of convoluted features yields better results than the original features to classify malignant and benign tumors.

**Abstract:**

Breast cancer is a common cause of female mortality in developing countries. Screening and early diagnosis can play an important role in the prevention and treatment of these cancers. This study proposes an ensemble learning-based voting classifier that combines the logistic regression and stochastic gradient descent classifier with deep convoluted features for the accurate detection of cancerous patients. Deep convoluted features are extracted from the microscopic features and fed to the ensemble voting classifier. This idea provides an optimized framework that accurately classifies malignant and benign tumors with improved accuracy. Results obtained using the voting classifier with convoluted features demonstrate that the highest classification accuracy of 100% is achieved. The proposed approach revealed the accuracy enhancement in comparison with the state-of-the-art approaches.

## 1. Introduction

According to World Health Organization (WHO) data, breast cancer is the sixth most prevalent cause of cancer mortality [1]. Breast cancer is a common malignancy that affects 2.1 million people globally every year [2]. In 2020, the mortality for breast cancer was 685,000, which made approximately 13.6% of all cancer deaths among women [2]. According to the statistics by Cancer Research UK (united kingdom), approximately 11,500 deaths are caused by breast cancer every year, indicating 32 deaths per day only in the UK [3]. Breast cancer is the second leading cause of mortality among women [4], which makes breast cancer one of the most lethal diseases in the present times. Malignant tumors cause breast cancer when cell growth becomes uncontrollable. Breast cancer develops when a large number of the breast’s fatty and fibrous tissues begin to grow abnormally. Cancer cells spread across tumors, resulting in different stages of cancer. As damaged cells and tissues spread throughout the body, breast cancer can express itself in a variety of ways [5]. Inflammatory breast cancer (IBC) is a kind of breast cancer that produces breast swelling and reddening. IBC is the fastest-growing type of breast cancer that occurs when the lymph arteries in the broken cell are blocked [6]. The second type is lobular breast cancer (LBC) [7], which grows within the lobule. It raises the chances of developing other invasive malignancies. Invasive ductal carcinoma (IDC) [8] is commonly known as infiltrative ductal carcinoma [9], and it is one of the most common types that are found in males. IDC grows in the breast tissues when abnormal breast cells grow. The fifth type of breast cancer is Mucinous breast cancer (MBC) [10], or colloid breast cancer; it is developed by the invasive ductal cells when abnormal tissues spread around the duct [11]. The non-invasive cancer is the Ductal carcinoma in situ (DCIS), which is usually developed when the abnormal cells move outside the breast [12]. The last type of breast cancer is mixed tumors breast cancer (MTBC), which is also known as invasive mammary breast cancer [11]. MTBC is developed by lobular cells and abnormal ducts.

Imaging techniques, physicians, and self-examination can all detect breast abnormalities. The biopsy is the only technique to determine whether or not there is cancer. For the early identification of breast cancer, various techniques such as ultrasound and mammography are available. Mammography is the most common and widely used screening method because of its high accuracy, high detectability, and low-cost [13]. Mammograms can be an excellent imaging technique for the classification and diagnosis of breast cancer with high accuracy. Nonetheless, mammography works poorly in some circumstances, particularly in patients with dense breast tissue. Furthermore, it has adverse effects related to severe ionized radiation in young women. However, it is a challenging task to observe lesions of a size smaller than 2mm using mammograms. Due to these limitations, mammography imaging is highly researchable for the early diagnosis of breast cancer [14].

Data mining is a useful process for extracting useful and meaningful information from the data. Data mining methods and functions help in the early detection of many diseases such as heart diseases [15], cancers, diabetes, leukemia, and lung cancer. In the conventional detection methodology, the detection of cancer is based on “the gold standard” technique that comprises three tests: physical examination, radiological imaging, and pathological tests. These methods are time-consuming, and the chance of a false-negative is still present. Contrary to traditional methods, machine learning methods are accurate, fast, and reliable. Recently, machine learning-based models have been utilized in disease detection, which assists medical experts to make a more accurate diagnosis. Such methods are efficient regarding disease detection, processing large amounts of data, reducing response time, etc. Keeping in view the potential of machine learning models, a machine learning-based approach is proposed for breast cancer detection, with an emphasis on providing high accuracy, making the following contributions.

- This study analyzes the impact of hand-crafted and deep convoluted features in breast cancer prediction. For convoluted features, this study uses the convolutional neural network (CNN).
- An ensemble model is proposed, which offers high breast cancer prediction accuracy. The model employs a logistic regression (LR) and stochastic gradient descent (SGD) classifier, and a voting mechanism is used to make the final prediction.
- Performance analysis is carried out by employing several machine learning models, including stochastic gradient descent (SGD), random forest (RF), extra tree classifier (ETC), gradient boosting machine (GBM), gaussian Naive Bayes (GNB), K-nearest neighbor (KNN), support vector machine (SVM), logistic regression (LR), and decision tree (DT). In addition, the performance of the proposed ensemble model is compared with the recent state-of-the-art models to show the significance of the proposed approach.

The organization of this paper is as follows: Section 2 briefly discusses the literature related to breast cancer detection and research gaps. Section 3 gives the proposed methodology along with the description of the ensemble model. Results are described in Section 4, while the conclusion of the study is given in Section 5.

## 2. Literature Review

This section of the study highlights the research gap in the field of breast cancer detection and classification. A considerable number of studies have been conducted in the domain of breast cancer detection. Computer-aided diagnostics (CAD) plays important role in the diagnosis of breast cancer in the preliminary stages. Different data mining techniques along with machine learning algorithms have a significant impact in this regard. In health analytics, it is very hard to analyze healthcare databases, as the data is vast and heterogeneous. Advancements in CAD and AI introduce accurate and precise systems for medical applications while dealing with medical data, which is sensitive in nature. Breast cancer is leading to a large number of deaths even in developed countries. Machine learning is extensively used in the diagnosis of breast cancer. Recently, many CAD and decision support have included studies for the detection of tumors, mainly breast cancer. To achieve accurate results, most of the studies use single techniques, while a few of them used ensemble models. This section of the study reviewed the most recent and state-of-the-art breast cancer detection techniques that employed machine learning.

Amrane et al. [16] compared KNN and Naive Bayes (NB) for the classification of breast cancer. The authors classified tumors into two benign or malignant classes. K-fold cross-validation is also applied to validate the performance. Experimental results show that KNN achieved 97.51% accuracy to perform binary classification. Obaid et al. [17] used machine learning algorithms for the classification of breast cancer. The authors compare the performance of three machine learning algorithms including SVM, KNN, and DT. SVM achieved an accuracy score of 98.1%. Nawaz et al. [18] performed multiclass classification by classifying tumors into three sub-classes. The authors applied CNN to the BreakHis dataset. The results demonstrate an accuracy of 95.4% using the deep CNN model on histopathology images.

Singh et al. [19] used auto-encoders for the prediction of breast cancer. For the detection of breast cancer, they used different machine-learning algorithms. They also proposed an auto-encoder model for the detection of breast cancer that works in an unsupervised manner. The authors worked on a compact feature representation that is strongly related to breast cancer. Auto-encoder outperformed the other classifiers used in the study and achieved a precision and recall score of 98.4%. The study by Allison Murphy [20] used the GFS-TSK for breast cancer diagnosis. Due to the capacity of the genetic algorithms, a fuzzy logic system gives a better representation of the dataset. For learning the optimal membership functions, a subset of data is used as the rule base of the fuzzy logic system. The ensemble of these two methods boosts the performance of cancer detection.

The study [21] proposed a machine learning-based system for the classification of breast cancer. The XGBoost is used with a different number of attributes. The reason for choosing the XGBoost for breast cancer prediction is that it is time efficient and more renowned for giving more precise results than other machine learning algorithms, when the number of features has reduced the accuracy of the XGBoost increases. On 30 features, the author achieved an accuracy of 97% while using 13 features, the achieved accuracy is 97.7%.

Akbulut et al. [22] performed the breast cancer classification using machine learning algorithms. The authors used three different machine learning models such as GBM, XGBoost, and LightGBM for breast cancer classification. The results of the study demonstrate that LightGBM outperforms the other machine learning models in terms of accuracy and achieved an accuracy score of 95.3%. On the Wisconsin breast cancer dataset, [23] used machine learning algorithms such as LR, DT, KNN, Naive Bayes (NB), RF, and rotation forest. The study implemented classification algorithms for three scenarios: all features were included, with highly correlated features included, and with low correlated features included. Results indicate that LR achieved the highest classification accuracy across all types of features.

Kashif et al. [24] proposed a hybrid model for breast cancer prediction through mammography images. They first segmented the mammogram images, then features were extracted using mammography processing. Afterwards, the mammography processing classification was conducted by using the extracted features. Entropy and texture features were used by Dey et al. [25] to extract the 112 features. Different machine learning algorithms such as KNN, SVM 1, SVM 2, and DT were used for the experiments. Results demonstrate an accuracy value of 78.9% using the manually extracted breast area.

An automatic breast cancer detection system using thermal images was proposed by Rajnikanth et al. [26]. The authors used two feature extraction pipelines including the local binary pattern (LBP) enhancement and feature extraction, and morphological segmentation, saliency enhancement, and GLCM features. Afterward, serial feature integrations are implemented. For the optimization of the features, the authors used Marine-predators algorithms (MPA). Different variants of SVM classifiers were also used to evaluate the optimized features. The overall achieved accuracy is 93.5%, which is obtained using SVM-cubic and SVM-coarse Gaussian. Hameed et al. [27] used two models, RetinaNet and you only look once (YOLO), for breast cancer recognition, and achieved an accuracy of 91%. The major drawback of their study is that they only used five mammogram image datasets. Abdar et al. [28] developed a two-layer nested ensemble (NE) model using stacking and voting techniques. They tested the proposed system on the same dataset used by [23] and achieved an accuracy of 98.07%.

Deep learning models have recently been developed for extracting features and enhancing the efficiency of the medical image analysis. Deep learning is a type of machine learning that employs multilayer convolution neural networks. Unlike other feature extraction methods, they have the ability to extract the features by themselves from the dataset directly. Convolution is used to extract the features from different parts of the image.

The study [29] used a transfer learning approach to design various CNNs. The study achieved an overall accuracy of 94.3%, recall of 93.3%, and precision of 94.7%. However, the study is limited by the fact that it is not using any segmentation technique to extract the breast area from other parts of the thermal images. Khan et al. [30] used pre-trained CNNs, including ResNet, GoogLeNet, and VGGNet, which were fed into the fully connected network layers for the classification of the cancerous benign cell by using average pooling classification. The study achieved an accuracy of 97.52%. McKinney et al. [31] proposed an AI-based system that outperformed human experts on breast cancer prediction using mammogram images. Tiney et al. [32] used mammogram images for the detection and classification of breast cancer and achieved a good accuracy and specificity of 90.50% and 90.71%, respectively. Barbosa et al. [33] used feature extraction techniques of the deep wavelet NN (DWNN). The study found that when the features are increased by adding additional levels in DWNN, better performance for the classification is achieved. The study achieved 79% specificity and 95% sensitivity. Despite the accuracy reported in the above-discussed research works, these works have the following limitations:

1. Several of these works used smaller datasets and the performance evaluation of the proposed approach is not evaluated properly,
2. Some of the previous works did not use breast area segmentation before the classification,
3. Many works include the manual region of interest extraction regarding the breast area,
4. Similarly, several works used the accuracy metric only. However, the good value of accuracy does not mean that the system can recognize different classes equally when an imbalanced dataset is used.

A comparative analysis of existing studies is presented in Table 1. Considering the above-stated shortcomings of existing literature, an automated approach is needed that can perform breast cancer detection automatically and with high accuracy. In addition, evaluation should be carried out considering several well-known performance evaluation metrics, such as accuracy, the area under the curve (AUC), sensitivity, specificity, etc.

## 3. Materials and Methods

**Figure 1:**
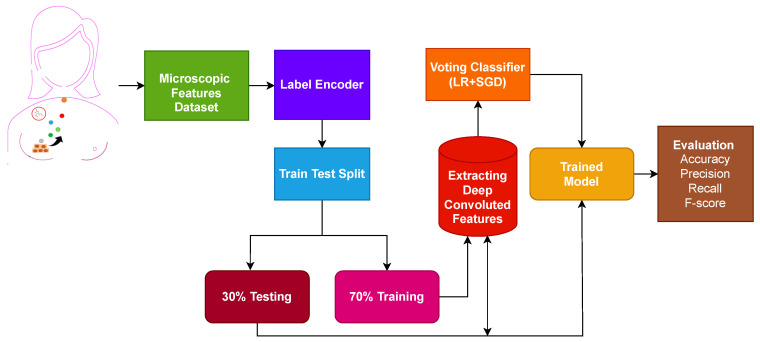
Workflow diagram of the adopted methodology.

In this section of the study, the proposed approach, the dataset used in this study, and the steps followed for the proposed approach are discussed. Figure 1 shows the workflow of the proposed approach.

The first step is data collection, where microscopic features related to the breast are extracted from the breast cell nuclei. The extracted features are preprocessed using a label encoder to convert categorical features into numeric form. The dataset contains no null values. Later, the processed microscopic features are divided into 70% training and 30% testing ratio using sklearn train-test validation. Deep convoluted features are used on the training set to obtain features.

**Figure 2:**
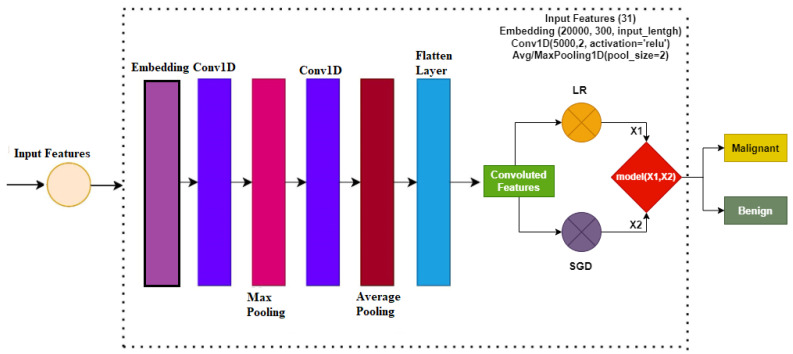
Proposed methodology architecture diagram.

Figure 2 shows the architecture of the proposed ensemble model. An ensemble voting classifier is proposed for breast cancer detection, which employs LR and SGD machine learning models. Instead of using hand-crafted features, a customized CNN is utilized for extracting prominent features from the dataset. These extracted features are then fed into LR and SGD for training. Voting is used on the output from these models to make the final prediction.

### 3.1. Dataset

Taking into account the performance of machine learning models, this work uses supervised machine learning models for breast cancer diagnosis. It proceeds through a series of activities, beginning with the dataset collection. This study makes use of the ’Breast Cancer Wisconsin Dataset’ from the UCI machine learning repository, which is freely available [34]. The dataset consists of 32 features. A brief dataset description is given in Table 2.

The dataset used in this study for breast cancer detection has two classes, which are ’benign’ and ’malignant’. The dataset contains 45% malignant and 55% benign samples. It consists of 32 attributes that are classified as numeric, nominal, binary, etc. A brief description of each attribute is given in Table 2. Out of 32 attributes, only the target attribute has categorical values, and the rest of the attributes belong to the numeric values.

### 3.2. Convolutional Neural Networks

In this study for the diagnosis of breast cancer, the CNN model is used for feature engineering. Such as other deep learning models, the CNN model has four layers, including the max-pooling layer, the embedding layer, the 1D convolutional layer, and the flatten layer. The first layer, which is the embedding layer, uses all the features from the breast cancer dataset with an embedding size of 20,000 and 300 output dimensions. The embedding layer is followed by the 1D convolutional layer with the 5000 filters. The 1D convolutional layer has an activation function ReLU (Rectified Linear Unit) and it has a kernel size of 2 × 2. For the significant feature map, a 2 × 2 max pooling layer is used from the output of the 1D convolution. in the end, flatten layer in the output is added to transform back to a 1D array for the machine learning model.

For instance, the breast cancer dataset consists of a tuple set ($fs_{i}$, $tc_{i}$), where fs represents the feature set and tc shows the column of the target class. The index of the tuple is denoted by *i*. For the conversion of the training set into the required input, the format embedding layer is used as:

(1) EL=embeddinglayer(Vs,Os,I)

(2) EOs=EL(fs)

where the output of the embedding layer is shown by $EO_{s}$. This embedding layer output is the input of the convolutional layer and EL shows the embedding layers. EL has three different parameters such as vocabulary size Vs, output dimensions Os, and input lengths *I*.

For breast cancer detection, the embedding size is set at 20,000, which means that the model can accept inputs between 0 to 20,000. Os are set at 300 and *I* as 32. The embedding layer processes the input data and creates output for the CNN model to process it further. Embedding layer output dimensions are EOs=(None,32,300):

(3) 1D−Convs=CNN(F,Ks,AF)←EOs

where 1D convolutional layer output is represented by 1D−Convs.

The output of the 1D convolutional layer is extracted from the embedding layer output. In this study, for the CNN, we used the 500 filters, i.e., F=500, and the kernel size of Ks=2×2. To set all the non-positive values to zero in the 1D−Convs output matrix, the ReLu activation function is used. ReLU only changed the only non-positive values to zero, while the rest of the values remained unchanged.

(4) f(x)=max(0,E)s

Max-pooling layer is used for the significant feature mapping from the CNN. For the feature set map, a pool of 2 × 2 is used. Where Fmap shows the features after the max-pooling, the stride is denoted by S−2, and Ps=2 is the size of the pooling window:

(5) $$Cf=Fmap=\lfloor \left(1-P_{s}\right)/S\rfloor +1$$

The flatten layer is used in the end to transform the 3D data into the 1D. The reason for this transformation is that it enhances the efficacy of the machine learning algorithms, as ML models work well on 1D data. By applying these steps, we obtained the 25,000 features for the machine learning models’ training.

### 3.3. Classifiers

Many classification algorithms can be investigated in conjunction with the extracted features to assess their performance. This study employs several of the most commonly used classification models. A brief description of each of these models is provided in Table 3.

### 3.4. Proposed Methodology

Widespread usage of ensemble models has increased the precision and effectiveness of categorization outcomes. When classifiers are combined, performance can be improved over time compared to using individual models. This study uses an ensemble learning approach to predict breast cancer in order to obtain better outcomes. The proposed method uses a voting classifier that combines LR and SGD, utilizing soft voting criteria. The end result will be the class with the highest voting score. Algorithm 1 explains the working of the proposed ensemble model, that can be expressed as:

(6) $$\hat{p}=argmax\left\{\sum_{i}^{n}LR_{i},\sum_{i}^{n}SGD_{i}\right\}.$$

**Figure 3:**
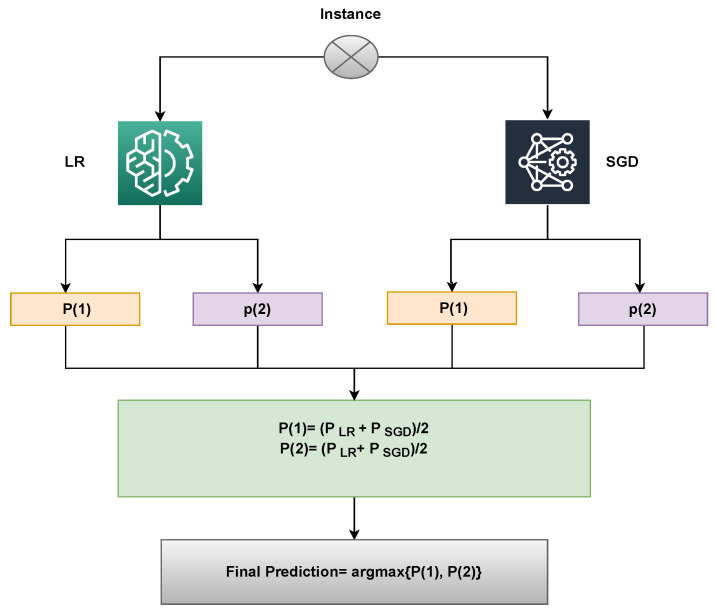
Architecture of the proposed voting classifier.

Here, $\sum_{i}^{n}LR_{i}$ and $\sum_{i}^{n}SGD_{i}$ both will provide prediction probabilities against each test sample. Following that, as shown in the figure, the probabilities for each test case using LR and SGD pass via the soft voting criterion Figure 3.

An illustration of the proposed approach’s capabilities can be used to describe it. Upon passing through the LR and SGD, a sample is supplied, and for each class, a probability score is given. Let Class 1 (Malignant) and Class 2 (Benign) have LR’s likelihood scores of 0.6 and 0.8, respectively. Class 1 (Malignant) and Class 2 (Benign) of SGD have probability scores of 0.8 and 0.9, respectively. Let P(x) be the probability score of x, and let x’s domain be constrained to the dataset’s four classes. The probability for the four classes may therefore be determined as follows:

P(1) = (0.6+ 0.8)/2 = 0.70

P(2) = (0.8+ 0.9)/2 = 0.85

The final prediction will be 2, whose probability score is the largest, as shown below:

(7) VC(LR+SGD)=argmax(g(x))

VC(LR+SGD) chooses the final class based on the maximum average probability of a class and combines the projected probabilities of both classifiers.
**Algorithm 1** Ensembling of LR and SGD.**Input:** input data ${\left(x,y\right)}_{i=1}^{N}$$M_{LR}$ = Trained_ LR$M_{SGD}$ = Trained_ SGD   1:**for**i=1toM**do**   2:      **if** $M_{LR}\neq 0\;\&\;M_{SGD}\neq 0\;\&\;training\_set\neq 0$ **then**   3:       $ProbSGD-1=M_{SGD}.probability\left(1-class\right)$   4:        $ProbSGD-2=M_{SGD}.probability\left(2-class\right)$   5:        $ProbLR-1=M_{RF}.probability\left(1-class\right)$   6:        $ProbLR-2=M_{RF}.probability\left(2-class\right)$   7:        Decision function = $max(\frac{1}{N_{classifier}}\sum_{classifier}$$\;(Avg_{\left(ProbSGD-1\;ProbLR-1\right)}$$\;,(Avg_{\left(ProbSGD-2,ProbLR-2\right)}$   8:      **end if**    9:     Return final label $\hat{p}$ 10:**end for** 

The proposed framework for breast cancer prediction is presented in Figure 3. The proposed VC(LR+SGD) is an ensemble of two machine-learning models. The breast Cancer Wisconsin dataset from the UCI repository was used in this experiment. First, the dataset is preprocessed by converting categorical values into the numerical form using a label encoder. The proposed model is applied to the Breast Cancer Wisconsin dataset in two phases. In the first phase, all 32 features of the dataset are used to predict breast cancer. In the second phase of the experiment, convoluted features are used to train all machine learning models and to predict cancerous patients. Then, the data was split into two parts, the training dataset, and testing data. The training data was given a percentage of 70%, while the testing data was 30%. The evaluation parameters used in this experiment are accuracy, precision, recall and F1 score.

### 3.5. Evaluation Metrics

The evaluation phase is a very important step of the study. In the evaluation phase, we evaluate the performance of the learning models. Several evaluation parameters are available for the evaluation of the learning models. This study uses renowned and commonly used evaluation parameters for breast cancer detection. These evaluation parameters are accuracy, precision, recall, and F1 score. All the matrices are based on the values provided in the confusion matrix. Classifier performance on the test data is elaborated using the confusion matrix. The evaluation parameters are computed using true positive (TP), true negative (TN), false positive (FP), and false negative (FN). The values of all the evaluation parameters used in this study range between 0 (min) and 1 (max).

Accuracy is a well-known and widely used parameter that is used to evaluate classifier performance. It is calculated using

(8) $$Accuracy=\frac{TP+TN}{TP+TN+FP+FN}$$

Precision and recall are other commonly used parameters for the classifier performance evaluation. Precision and recall considers the positive cases and can be calculated as:

(9) $$Precision=\frac{TP}{TP+FP}$$

(10) $$Recall=\frac{TP}{TP+FN}$$

Out of all the aforementioned matrices, the F1 score has been regarded as the most important metric. F1 score is commonly used for classification problems, and it is a statistical measure. It is the mean of the precision and recall and its values range from 0 to 1. Mathematically, it is calculated as:

(11) $$F1-Score=2\times \frac{Precision\times Recall}{Precision+Recall}$$

## 4. Experiments and Results

This paper conducts several experiments to compare the performance of the proposed methodology to different machine learning and deep learning models. All experiments are conducted on an Intel Corei7 7th generation computer with Windows 10. TensorFlow, Keras, and Sci-kit Learn frameworks in Python are used to implement the proposed technique as well as machine learning and deep learning models. Experiments are conducted independently, with both the original feature set from the breast cancer dataset and the CNN features used.

### 4.1. Performance of Models Using Original Features

Firstly, the experiments are performed with the original feature set from the breast cancer dataset. Table 4 shows the results of all classifiers using original features. The results demonstrate that the proposed voting ensemble model LR+SGD performs better than all other models with a significant accuracy of 0.772. Similarly, LR and SGD classifiers also achieved good accuracy scores of 0.769 and 0.761, respectively. Tree-based ensemble model ETC achieved an accuracy value of 0.759. Tree-based model RF achieved the least accuracy of 0.743 among all models. However, the ensemble of linear models (LR+SGD) shows better performance on the original feature set.

The voting ensemble model performance is good when it is compared with the linear models. The main factor behind this performance is that the voting model works well with a large feature set. LR and SGD individually performed well and the ensemble of them boost the performance. Although the ensemble model performs well, the achieved accuracy falls short of the requirements for breast cancer diagnosis and needs to be improved. Further experiments are carried out for this proposal using the CNN extracted features and an ensemble machine-learning model.

### 4.2. Performance of Models Using CNN Features

The results of the second set of experiments, which used CNN features to analyze the performance of machine learning and the proposed ensemble model, are shown in Table 5. The objective of using CNN model features is to expand the feature set, which is expected to improve linear model accuracy. Machine learning models are trained and tested using CNN-extracted features.

The experimental results reveal that the proposed voting ensemble model LR+SGD outperforms all other models, achieving the highest accuracy of 1.00. It shows a significant increase in the performance of LR+SGD and an improvement of 0.228 in the performance over the original features. Similarly, as compared to the original feature set, the individual linear models performed better with the CNN features. LR achieved an accuracy of 0.991 while the SGD obtained an accuracy value of 0.986; these results demonstrate that the improvement in their accuracy is 0.222 and 0.225, respectively. GBM and tree-based classifier RF achieved the least accuracy value of 0.951 on the CNN features. The number of features increases significantly when CNN is used for feature extraction, resulting in a significant improvement in model performance. Linear models outperform other models because the features generated by the CNN model are highly correlated with the target class and make the data linearly separable.

### 4.3. Results of K-Fold Cross-Validation

K-fold cross-validation is used to verify the effectiveness of the models. The complicated aspects of the suggested technique are utilized for this. Table 6 provides the results of the 10-fold cross-validation. It indicates that the performance of the proposed approach is superior regarding the accuracy, precision, recall, and F1 score with a small standard deviation.

### 4.4. Performance Comparison with Existing Studies

To corroborate the performance of the proposed approach, a performance comparison is carried out with the existing state-of-the-art models that investigate breast cancer detection. For this purpose, several recent studies from the literature are selected. For example, [42] uses PCA features with an SVM model for cancer detection and shows a 96.99% accuracy. An auto-encoder is used in [19] to obtain a 98.40% accuracy. The study [17] employs quadratic SVM and achieves a 98.11% accuracy. An XgBoost is used in [21] for the same task, which obtains a 97.11% accuracy score. Similarly, [23,43] obtains 98.21% and 98.10% accuracy scores, respectively, by utilizing Chi-square features and LR with all features, respectively. Despite the high accuracy reported in these research works, the proposed models demonstrate better results, as shown in Table 7. The acronyms used in the manuscript are given in Table 8.

### 4.5. Statistical t-Test

The importance of the suggested technique has also been demonstrated using the statistical *T*-Test. In the *T*-test, the null hypothesis Ho indicates that the accuracy difference between approaches is not significant, but the alternate hypothesis Ha indicates that the accuracy difference is significant. We have tested the proposed model against the top-performing model from the earlier research [19]. The test yields a result of 9.22158 for test statistics and a *p*-value of 0.001349. It is concluded that the performance has improved as a result of the suggested model. Results demonstrate that the difference has a *p* 0.05 value, which is statistically significant. The suggested model scored the top on accuracy in terms of mean rank.

### 4.6. Limitations of Study

The limitation of this study is that the dataset was gathered from a single source. Because of this, it is not possible to generalize the results according to the multicenter research. The advantage of this study over previous studies is that the significant features are extracted using CNN. Thus, risk factors for breast cancer have been identified that may be significant.

## 5. Conclusions

The motivation of this research work is to develop a framework that accurately classifies malignant and benign patients, and reduces the risk associated with this leading cause of death in women. In research related to human healthcare, accuracy is considered the most important factor. The proposed approach aims at increasing the accuracy while minimizing the prediction error for breast cancer. Experimental results indicate that using convolutional features tends to obtain a higher accuracy than the original features. Moreover, the ensemble classifier of LR and SGD shows better performance than individual models. Performance comparison with the state-of-the-art studies shows the superior performance of the proposed approach. Again, the higher accuracy as compared to other approaches shows the effectiveness of this framework. In the future, we intend to perform cancer-type classifications with convoluted features using deep-learning ensemble models. This study uses a dataset collected from a single source. In the future, we intend to apply the proposed approach to other datasets to prove its generalizability.

## Figures and Tables

**Table 1:** Comparative analysis of the existing approaches.

Ref.	Methods	Dataset	Findings
[16]	KNN and NB	Breast cancer dataset	KNN achieved 97.5% accuracy.
[17]	SVM, KNN and DT	Breast cancer dataset	High performance by SVM with 98.1% accuracy.
[18]	CNN	BreakHis dataset	CNN achievd 95.4% accuracy.
[19]	Auto encoder	Breast Cancer Wisconsin dataset	The proposed approach surpassed other models.
[20]	Fuzzy Logic Systems	Breast Cancer Wisconsin dataset	Genetic algorithm outperforms in combination with fuzzy logic.
[21]	XGBoost	Breast Cancer Wisconsin dataset	The proposed approach achieved 977% accuracy using 13 features.
[22]	GBM, XGBoost, LightGBM	Breast Cancer Wisconsin dataset	LightGBM has shown robust results in breast tumor classification.
[23]	LR, DT, KNN and NB	Breast Cancer Wisconsin dataset	LR achieved highest results.
[24]	ML algorithms	Mammogram	Hybrid models have shown robust results
[25]	KNN, SVM and DT	Breast thermal images	firefly algorithm in applied to improve the quality of images.
[26]	SVM-cubic and SVM-coarse Gaussian	Breast thermal images	The proposed apptoach achieved 93.5% accuracy.
[27]	RetinaNet, YOLO	Mammogram	The study is limited in way that it uses only five images in experiments.
[28]	NE Model	Breast Cancer Wisconsin dataset	The proposed layered ensemble approach outperformed other individual models.
[29]	Transfer learning with CNN	Thermal images	The proposed approach has achieved 94.3% accuracy.
[30]	GoogleNet, VGGNet, ResNet	breast microscopic image data sets	The proposed transfer learning approach surpassed the individual models.
[31]	AI-based system	Mammogram	The proposed approach have shown promising results.
[32]	CNNI-BCC	Mammogram	The proposed approach used CNN to improve breast cancer classification.
[33]	Deep-wavelet neural network (DWNN)	Thermographic images	The proposed model detected breast lesion with 95% sensitivity.

**Table 2:** Dataset features and their description.

Feature	Description
id	It represents the ID number of the.
fractal_dimension_worst	It is the “worst” or largest mean value for “coastline approximation” −1
radius_mean	It is the mean of the distance from the center to its perimeter.
concave points_worst	It is the "worst" or largest mean value for the number of concave portions of the contour.
perimeter_mean	It is the mean of core tumor size.
compactness_worst	It is the “worst” or largest mean value for perimeter⌃2 / area −1.0
smoothness_mean	It is the mean of local variation in radius lengths
fractal_dimension_mean	It is the mean for “coastline approximation” −1
concavity_mean	It is the mean of the severity of concave portions of the contour
texture_se	It is the standard error for standard deviation of gray-scale values
symmetry_mean	
concave points_mean	It is the mean for the number of concave portions of the contour
radius_se	It is the standard error for the mean of distances from the center to points on the perimeter
texture_worst	It is the "worst" or largest mean value for standard deviation of gray-scale values
perimeter_se	
perimeter_worst	
smoothness_se	It is standard error for local variation in radius lengths
radius_worst	It is the “worst” or largest mean value for the mean of distances from the center to points on the perimeter
concavity_se	It is the standard error for the severity of concave portions of the contour
symmetry_se	
fractal_dimension_se	It is the standard error for “coastline approximation” −1
concave points_se	It is the standard error for the number of concave portions of the contour
area_se	
smoothness_worst	It is the “worst” or largest mean value for local variation in radius lengths
area_worst	
compactness_se	It is the standard error for perimeter⌃2 / area −1.0
compactness_mean	It is the mean of perimeter⌃2 / area −1.0
concavity_worst	It is the “worst” or largest mean value for the severity of concave portions of the contour
area_mean	
symmetry_worst	
texture_mean	It is the standard deviation of gray-scale values
Diagnosis (Target Class)	The diagnosis of breast tissues (M = malignant, B = benign)

**Table 3:** Brief description of machine learning models.

MLA	Description	Advantages	Limitations
RF [35]	For the development of the decision trees. It performed a random selection of features with controlled variance.	Decreased in overfitting.	Very slow in real-time prediction. Complex classifier
KNN [36,37]	It is a straightforward instance-based classifier widely used in medical data mining.	The optimal value is easily achieved through it.	Classification is very slow.
DT [38]	From the set of class labels, it constructs the decision trees. It is a structural method represented as a flow chart similar to a tree.	It combines numeric and categorical data. Very fast and simple.	Problems with the high dimensionalities and unbalanced data. A longer training time is needed. Not a good choice for larger datasets.
SVM [39]	It is a linear classification algorithm that works well on low-dimensional and uncomplicated data. However, it also gives good results on complex and high-dimensional data.	Easily separate the data space. One of the most robust and accurate algorithms. Has a strong basis in statistical learning theory.	Classification is very slow. Required longer training time.
LR [40]	It is a linear model for classification rather than regression. It uses the regression model to estimate the probability of the class members.	More robust and handles nonlinear data. Good for numeric and categorical classification.	Boolean values only. Not a good choice for predicting the value of a binary value.
GBM [41]	In conjunction, it enhances the classifier performance. Very sensitive to handling noisy data.	Less suspectable to overfitting problems.	Very sensitive to outliers. Pre-adjustment is needed to achieve optimal performance.

**Table 4:** Performance of machine learning models using the original features.

Model	Accuracy	Class	Precision	Recall	F1 Score
Voting (LR + SGD)	0.772	B	0.75	0.79	0.77
		M	0.78	0.80	0.79
		M Avg.	0.76	0.79	0.78
		W Avg.	0.76	0.79	0.78
GBM	0.745	B	0.77	0.81	0.79
		M	0.80	0.81	0.80
		M Avg.	0.79	0.81	0.79
		W Avg.	0.78	0.81	0.79
GNB	0.756	B	0.78	0.82	0.80
		M	0.80	0.80	0.80
		M Avg.	0.79	0.81	0.80
		W Avg.	0.79	0.80	0.80
ETC	0.759	B	0.78	0.83	0.80
		M	0.82	0.85	0.83
		M Avg.	0.80	0.84	0.81
		W Avg.	0.80	0.85	0.82
LR	0.769	B	0.79	0.79	0.79
		M	0.82	0.83	0.82
		M Avg.	0.81	0.81	0.80
		W Avg.	0.80	0.81	0.80
SGD	0.761	B	0.80	0.80	0.80
		M	0.83	0.81	0.82
		M Avg.	0.81	0.80	0.81
		W Avg.	0.81	0.80	0.81
RF	0.743	B	0.72	0.73	0.72
		M	0.77	0.85	0.81
		M Avg.	0.75	0.79	0.77
		W Avg.	0.75	0.80	0.77
KNN	0.751	B	0.78	0.82	0.80
		M	0.81	0.83	0.82
		M Avg.	0.79	0.82	0.81
		W Avg.	0.79	0.81	0.81
SVM	0.767	B	0.77	0.79	0.78
		M	0.80	0.81	0.80
		M Avg.	0.78	0.80	0.79
		W Avg.	0.78	0.80	0.79
DT	0.739	B	0.70	0.71	0.70
		M	0.74	0.78	0.76
		M Avg.	0.72	0.74	0.73
		W Avg.	0.72	0.74	0.73

**Table 5:** Models’ performance using convoluted features from CNN.

Model	Accuracy	Class	Precision	Recall	F1 Score
Voting (LR + SGD)	1.000	B	1.00	1.00	1.00
		M	1.00	1.00	1.00
		M Avg.	1.00	1.00	1.00
		W Avg.	1.00	1.00	1.00
GBM	0.951	B	0.94	0.98	0.96
		M	0.96	0.91	0.94
		M Avg.	0.95	0.95	0.95
		W Avg.	0.95	0.95	0.95
GNB	0.965	B	0.98	0.96	0.97
		M	0.95	0.97	0.96
		M Avg.	0.96	0.97	0.96
		W Avg.	0.97	0.97	0.97
ETC	0.965	B	0.95	0.99	0.97
		M	0.98	0.93	0.96
		M Avg.	0.97	0.96	0.96
		W Avg.	0.97	0.97	0.96
LR	0.991	B	0.99	1.00	0.99
		M	1.00	0.98	0.99
		M Avg.	0.99	0.99	0.99
		W Avg.	0.99	0.99	0.99
SGD	0.986	B	0.98	1.00	0.99
		M	1.00	0.97	0.98
		M Avg.	0.99	0.98	0.99
		W Avg.	1.00	0.97	0.98
RF	0.951	B	0.94	0.98	0.96
		M	0.96	0.91	0.94
		M Avg.	0.95	0.95	0.95
		W Avg.	0.95	0.95	0.95
KNN	0.972	B	0.98	0.95	0.96
		M	0.95	0.92	0.94
		M Avg.	0.96	0.94	0.95
		W Avg.	0.96	0.93	0.95
SVM	0.979	B	0.97	0.93	0.95
		M	0.95	0.92	0.93
		M Avg.	0.96	0.92	0.94
		W Avg.	0.96	0.92	0.94
DT	0.943	B	0.92	0.90	0.91
		M	0.93	0.91	0.92
		M Avg.	0.92	0.90	0.91
		W Avg.	0.92	0.90	0.91

**Table 6:** 10-fold cross-validation results for proposed approach.

Fold Number	Accuracy	Precision	Recall	F-Score
Fold-1	0.999	0.999	0.999	0.999
Fold-2	0.996	0.997	0.998	0.997
Fold-3	0.999	0.999	0.999	0.999
Fold-4	1.000	1.000	1.000	1.000
Fold-5	1.000	1.000	1.000	1.000
Fold-6	1.000	1.000	1.000	1.000
Fold-7	0.997	0.996	0.998	0.997
Fold-8	0.999	0.999	0.999	0.999
Fold-9	0.999	0.999	0.999	0.999
Fold-10	1.000	1.000	1.000	1.000
**Average**	**0.9989**	**0.9989**	**0.9992**	**0.9990**

**Table 7:** Performance comparison with state-of-the-art studies.

Reference	Approach	Accuracy
[44]	K-means clustering	92.01%
[42]	PCA features with SVM	96.99%
[17]	Quadratic SVM	98.11%
[19]	Auto-encoder	98.40%
[20]	GF-TSK	94.11%
[21]	XgBoost	97.11%
[22]	Five most significant features with LightGBM	95.03%
[43]	Chi-square features	98.21%
[23]	LR with all features	98.10%
**Proposed**	Deep convoluted features with voting classifier (LR + SGD)	100%

**Table 8:** The acronyms used in this manuscript.

Acronyms	Definition
AI	Artificial Intelligence
AUC	Area under the curve
CAD	Computer-aided diagnostic
CNN	Convolutional Neural Network
DCIS	Ductal carcinoma in situ
DWNN	deep wavelet Neural network
DT	Decision Tree
ETC	Extra Tree classifier
GBM	Gradient boosting machine
GNB	Gausssian Naive Bayes
IBC	Inflammatory breast cancer
IDC	Invasive ductal carcinoma
LBC	Lobular breast cancer
KNN	K nearest neighbor
LR	Logistic Regression
MBC	Mucinous breast cancer
MTBC	Mixed tumors breast cancer
PCA	Principal component analysis
ReLU	Rectified Linear Unit
SGD	Stochastic gradient descent
SVM	Support vector machine
VC	Voting classifier
WHO	World health organization

## Data Availability

Not applicable.
